# Allocation of cognitive resources in cognitive processing of rhythmic visual stimuli before gait-related motor initiation

**DOI:** 10.3389/fnins.2023.1145051

**Published:** 2023-05-12

**Authors:** Huilin Zhou, Wenfeng Yan, Jialin Xu, Yehao Ma, Guokun Zuo, Changcheng Shi

**Affiliations:** ^1^Ningbo Institute of Materials Technology and Engineering, Chinese Academy of Sciences, Ningbo, Zhejiang, China; ^2^Ningbo Cixi Institute of Biomedical Engineering, Ningbo, Zhejiang, China; ^3^Zhejiang Engineering Research Center for Biomedical Materials, Ningbo Institute of Materials Technology and Engineering, Chinese Academy of Sciences, Ningbo, Zhejiang, China; ^4^Faculty of Electrical Engineering and Computer Science, Ningbo University, Ningbo, Zhejiang, China; ^5^Robotics Institute, Ningbo University of Technology, Ningbo, Zhejiang, China

**Keywords:** rhythmic visual stimuli, electroencephalography, allocation of cognitive resources, gait-related motor initiation, motor preparation

## Abstract

Rhythmic visual cues can affect the allocation of cognitive resources during gait initiation (GI) and motor preparation. However, it is unclear how the input of rhythmic visual information modulates the allocation of cognitive resources and affects GI. The purpose of this study was to explore the effect of rhythmic visual cues on the dynamic allocation of cognitive resources by recording electroencephalographic (EEG) activity during exposure to visual stimuli. This study assessed event-related potentials (ERPs), event-related synchronization/desynchronization (ERS/ERD), and EEG microstates at 32 electrodes during presentation of non-rhythmic and rhythmic visual stimuli in 20 healthy participants. The ERP results showed that the amplitude of the C1 component was positive under exposure to rhythmic visual stimuli, while the amplitude of the N1 component was higher under exposure to rhythmic visual stimuli compared to their non-rhythmic counterparts. Within the first 200 ms of the onset of rhythmic visual stimuli, ERS in the theta band was highly pronounced in all brain regions analyzed. The results of microstate analysis showed that rhythmic visual stimuli were associated with an increase in cognitive processing over time, while non-rhythmic visual stimuli were associated with a decrease. Overall, these findings indicated that, under exposure to rhythmic visual stimuli, consumption of cognitive resources is lower during the first 200 ms of visual cognitive processing, but the consumption of cognitive resources gradually increases over time. After approximately 300 ms, cognitive processing of rhythmic visual stimuli consumes more cognitive resources than processing of stimuli in the non-rhythmic condition. This indicates that the former is more conducive to the completion of gait-related motor preparation activities, based on processing of rhythmic visual information during the later stages. This finding indicates that the dynamic allocation of cognitive resources is the key to improving gait-related movement based on rhythmic visual cues.

## 1. Introduction

A large number of experimental studies have shown that rhythmic visual cues can improve gait initiation (GI) among patients with freezing of gait (FOG) in Parkinson's disease (PD) (Lim et al., 2005; Azulay et al., 2006; Spaulding et al., 2013; Lu et al., 2017). However, the neurophysiological mechanisms underlying the role of rhythmic visual cues in gait improvement is unclear. Muralidharan et al. (2017) showed that conflicting visual cues can affect GI among patients with PD. They proposed that visual cues with low cognitive load can alleviate FOG, while visual cues with high cognitive load can induce FOG. According to the theory of cognitive load, limited cognitive resources may be depleted under a task with a high cognitive load, resulting in a decline in motor performance (Chen et al., 2018). Not only are external sensory cues considered to improve gait control, they are also believed to decrease the cognitive processing demands of gait control for patients with PD (Rochester et al., 2007). Therefore, sensorimotor processing of visual cues may affect the use of cognitive resources in patients with PD, especially when these patients cannot use external cues to guide their own movements (Pieruccini-Faria et al., 2014). These findings suggest that the modulating effect of rhythmic visual cues on FOG may be related to level of cognitive load and the allocation of cognitive resources. Therefore, analysis of the influence of rhythmic visual cues on the allocation of cognitive resources during gait-related motor initiation may represent an opportunity for a breakthrough in understanding the regulatory mechanism of FOG.

In a previous study, we took the lead in comparing the effects of rhythmic visual cues and those of non-rhythmic visual cues on the allocation of cognitive resources during GI, focusing mainly on the motor preparation stage of GI. The results of this comparison have shown that the motor preparation occurs approximately 1,500 ms before motor imperative stimuli; under rhythmic visual cues, the demand for allocation of cognitive resources during motor preparation of GI is lower compared to demand under their non-rhythmic counterparts (Zhou et al., 2021). Therefore, we hypothesize that exposure to rhythmic visual cues is more conducive in FOG patients to the use of their gradually depleted dopaminergic energy for allocation of cognitive resources and achievement of motor preparation and completion of GI.

In the process of converting visual information to motor planning, the input of visual information plays a key guiding role in motor preparation (Jahfari et al., 2015). In the batting preparation process of professional athletes, the time resolution of visual perception is very high, and variations in processing speed would affect their performance (Hagura et al., 2012). Other studies have shown that visual and auditory stimuli can produce anticipatory postural adjustments before GI and exert a rapid effect on the use of preparation strategies (Watanabe et al., 2016). However, it is not clear how the input of rhythmic visual information modulates the allocation of cognitive resources and affects the motor preparation stage of GI. The characteristics of the spatiotemporal distribution of cognitive resources in perception of rhythmic visual stimuli before motor preparation need to be further studied.

As a wearable brain imaging technology with high time resolution, electroencephalography (EEG) has developed into a reliable tool for the study of cognitive processes in the cerebral cortex in recent years; this tool offers the possibility of analyzing neurodynamic modulation in the effect of rhythmic visual cues on gait (Lyu et al., 2017). Previous studies have shown that analysis of the time-domain characteristics of event-related potentials (ERPs) and the time–frequency characteristics of event-related synchronization/desynchronization (ERS/ERD), measured via EEG, is an effective method of characterizing cognitive resources (Yu et al., 2020; Stuart et al., 2021). The distribution of ERPs is dependent largely on the types and physical parameters of the corresponding stimuli. In the early period, as an important component of visual ERPs, C1 is highly sensitive to contrast and spatial frequency in visual stimuli. The polarity of its amplitude is related to stimulation of the upper vs. lower visual fields (Luck et al., 2000). Additionally, the N1 component, which is closely related to the initial processing of a stimulus, can represent the demands of early perceptual processing (Hickey et al., 2020; Chen et al., 2022a,b). Finally, the P2 and P3 components of the ERP, representing the response to visual stimuli over the parietal region, are the most common indices for evaluation of cognitive resources (Yang et al., 2020; Reiser et al., 2021; Vila-Cha et al., 2021). The higher the amplitude of these components, the higher the consumption of cognitive resources is generally considered to be.

In the time–frequency domain, ERS/ERD in the theta, alpha, and beta frequency bands reflect the oscillation of cortical activation (Yu et al., 2020), representing readiness to process information (Tard et al., 2016). The occurrence of beta oscillation is influenced by the demands of attentional tasks (Delval et al., 2020). Velu et al. (2014) found that visual cues can significantly enhance beta-band information flow in the occipital–parietal–motor cortex network in patients with FOG; this finding indicates that visual cues effectively activate other neural pathways to alleviate FOG symptoms.

In addition to traditional approaches to EEG analysis, in recent years, microstate analysis has been introduced as a new method to further study neurodynamics (Duc and Lee, 2019; Pal et al., 2021). The spatial configuration (i.e., the topography) of the global electric field of the EEG/ERP signal recorded on the scalp during a cognitive task does not change at every recorded time point: it remains stable over periods of several tens of milliseconds and then shifts rapidly into a different stable topographic configuration, which again remains stable for a certain period before changing again (Laganaro, 2017). These periods of a quasi-stable global electric field over the scalp are directly linked to the configuration of the intracranial sources and likely reflect specific periods in the mental processing of information (Britz et al., 2014).

To the best of our knowledge, no study has reported on changes in EEG microstate during cognitive processing of rhythmic visual stimuli. In this study, traditional ERP and ERS/ERD analysis was applied, in combination with use of the microstate analysis method, to study the spatiotemporal allocation of cognitive resources in the cognitive processing of rhythmic visual stimuli.

## 2. Methods

### 2.1. Participants

A total of 20 healthy subjects participated in this study voluntarily. All of them reported having normal or corrected-to-normal vision and reported no history of neurological diseases or lower limb disorders. All experimental procedures were conducted in accordance with the Declaration of Helsinki. Before the experiment, all participants signed a written statement of informed consent approved by the local Research Ethics Committee. Upon pre-processing analysis of the electroencephalogram (EEG) signal, we found that two participants blinked frequently during the task, exceeding the normal rate of 15~20 times per min, resulting in excessive eye movements and artifacts. To avoid the possibility of this affecting the quality of the data, we excluded the data from these two participants. Therefore, EEG data from 18 participants (15 men, 3 women; mean age: 27.0 years, SD: 4.0) were included in the final analysis.

### 2.2. Experimental procedures

**Figure 1:**
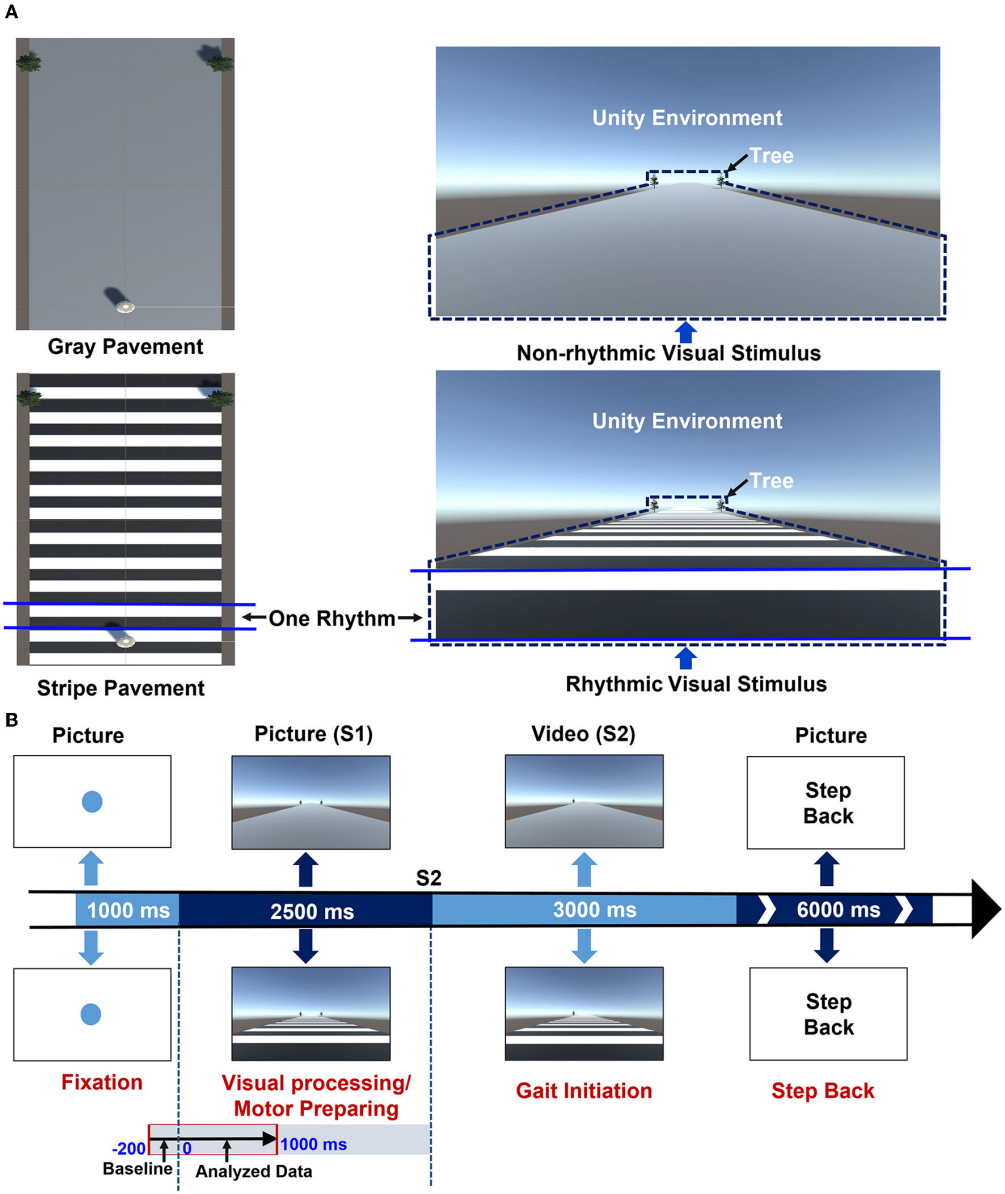
The experimental design. **(A)** A schematic diagram of the non-rhythmic and rhythmic visual stimuli. The non-rhythmic visual stimulus consisted of a gray pavement, while the rhythmic visual stimulus consisted of a pavement with equidistant black-and-white stripes. For the rhythmic visual stimulus, each black or white stripe was considered to represent one piece of rhythmic information. In addition, in both visual stimuli, trees were presented as reference objects to simulate a dynamic walking scenario. **(B)** A schematic diagram of the experimental design for each trial. Each trial consists of four phases. In the first phase (1,000 ms), a blue fixation point was presented, reminding participants to maintain a standing position. In the second phase (2,500 ms), a stationary visual cue was presented, either non-rhythmic (upper image) or rhythmic (lower image), reminding participants to prepare for GI. In the third phase (3,000 ms), a video was presented with the same type of visual cue, prompting participants to complete GI according to the prompts. In the final phase (6,000 ms), the instruction “Step Back” was presented to prompt participants to step back to the starting point. The second phase consisted of two stages: cognitive processing of visual stimuli (approx. 1,000 ms) and motor preparation for GI (approx. 1,500 ms). This study focused on the entire visual cognitive processing stage.

The experimental paradigm was designed as described in a previous article by Zhou et al. (2021). All stimulus paradigms were programmed in E-Prime 2.0 and presented on a 65-inch screen. Non-rhythmic and rhythmic visual stimuli were designed by Unity Personal (Unity Technologies Inc, San Francisco, USA). As shown in Figure 1A, the non-rhythmic visual stimulus consisted of a gray pavement, while the rhythmic visual stimulus consisted of a pavement with equidistant black-and-white stripes. In the case of the rhythmic visual stimulus, each black or white stripe was considered to be one piece of rhythmic information. In addition, at the horizon of the visual stimulus, trees were presented as reference objects to simulate dynamic walking conditions. The experimental tasks consisted of six blocks: three blocks in which GI was triggered by non-rhythmic visual stimuli, and three blocks in which GI was triggered by rhythmic visual stimuli. Participants were randomly assigned to complete either the non-rhythmic blocks or the rhythmic blocks first. There were 40 trials in each block, and each trial consisted of four phases, as shown in Figure 1B: fixation, GI anticipation (visual processing and motor preparing), GI response, and stepping back.

In the first phase (1,000 ms), a blue fixation point was presented, reminding participants to maintain a standing position. In the second phase (2,500 ms), a stationary visual cue was presented, either non-rhythmic (upper image) or rhythmic (lower image), reminding participants to prepare for GI. In the third phase (3,000 ms), a video was presented with the same type of visual cue, prompting participants to complete GI according to the prompts. In the final phase (6,000 ms), the instruction “Step Back” was presented to prompt participants to step back to the starting point. All participants were asked to complete the corresponding tasks as prompted by the external visual cues presented on the screen. All experimental tasks were conducted in an electromagnetic shielding room to reduce disturbance from the environment.

### 2.3. EEG acquisition and preprocessing

EEG data from 32 active monopolar Ag/AgCl electrodes (Fp1, Fpz, Fp2, AF3, AF4, F3, F1, FZ, F2, F4, FC3, FC1, FCZ, FC2, FC4, C5, C3, C1, CZ, C2, C4, C6, CP3, CP1, CPZ, CP2, CP4, P3, P1, PZ, P2, and P4) were recorded using SynAmps-2 amplifiers and Neuroscan CURRY^®^ Neuroimaging acquisition software (Neuroscan, El Paso, USA). The electrodes were mounted in a 64-channel electrode cap (Quick-Cap, Neuroscan, El Paso, USA), with their distribution conforming to the extended International 10–20 system. Two additional monopolar electrodes were placed at the left and right mastoids (M1 and M2). Two bipolar electrode pairs were used to record vertical and horizontal electrooculography (EOG) for detection of eye movements and blinks. EEG data were collected via digital acquisition, and the filtering range varied from 0 to 400 Hz using a 24-bit A/D converter. The sampling rate was set to 1,000 Hz. Additionally, the impedance of each electrode was below 10 kΩ for all participants.

EEG data were pre-processed offline using EEGLAB, an open-source toolbox running in the MATLAB environment (Delorme and Makeig, 2004). The raw data were filtered with a band-pass filter over the range of 0.1–30 Hz and a notch filter at 50 Hz. All EEG signals were subsequently re-referenced to the average of the left and right mastoids. As shown in Figure 1B, the second phase of each trial consisted of two stages: cognitive processing of the visual stimulus (approximately 1,000 ms) and motor preparation for GI (approximately 1,500 ms). This study focused on the entire visual cognitive processing stage. Therefore, the continuous EEG recording was segmented into epochs of 1,200 ms, each ranging from −200 to +1000 ms relative to the onset of the individual stimulus in the GI anticipation phase of the trial. All EEG epochs were baseline-corrected in the time domain using the −200 to 0 ms epoch as a baseline. Eye movement and motion artifacts were removed via an Independent Component Analysis (ICA) procedure; artifact-free epochs of EEG signal with voltages in the range ±100 μV were retained.

### 2.4. EEG analysis

#### 2.4.1. ERP analysis

Event-related potentials were calculated by segmenting data into post-stimulus epochs of 1,000 ms following onset of the GI anticipation phase and averaging voltages across trials separately for each participant and condition. We obtained an average of 106 artifact-free segments per participant under the non-rhythmic condition and 103 artifact-free segments per participant under the rhythmic condition. Based on previous literature on visual research, we calculated voltages for four ERP components over parietal electrode sites. Specifically, we calculated voltages for C1, N1, P2, and P3 at electrode PZ for each participant for each experimental condition. P1/C1 was quantified as the mean voltage in the time window ± 15 ms either side of the peak (with the peak occurring at 120–150 ms); N1 as the mean voltage in the time window ± 20 ms either side of the peak (180–220 ms); P2 as the mean voltage in the time window ± 10 ms either side of the peak (270–290 ms); and P3 as the mean voltage ± 15 ms either side of the peak (375–405 ms). For each condition, the grand average ERP values were calculated by averaging data across participants.

#### 2.4.2. Microstate analysis

For the microstate analysis, multichannel EEG signals were regarded as sequences of instantaneous topographic maps of the electric field. To further interpret and identify the differences in ERP components under different visual cues, EEG microstate analyses were performed using the CARTOOL software (Brunet et al., 2011). Mathematical clustering using the Topographic Atomize and Agglomerate Hierarchical Clustering (T-AAHC) method was conducted as the first step. The topographic map at peak global field power (GFP) and its spatial correlation coefficient with the initial category were used as the basis for microstate category recognition. The clusters were not labeled if the spatial correlation coefficient was below 50%. Some temporal post-processing was performed in the second step. If the correlation between the two segments was above 95%, the two segments were combined into a single segment. Small segments shorter than or equal to 20 ms were rejected. The optimum number of clusters between the non-rhythmic and rhythmic conditions was calculated using the cross-validation (CV) criterion in the final step.

#### 2.4.3. ERS/ERD analysis

To measure mean event-related changes in the power spectrum, event-related spectral perturbation (ERSP) analysis was conducted by time–frequency decomposition (TFD) using the short-time Fourier transform (STFT) with a fixed Hanning window of 200 ms. For each trial, the time–frequency estimate at each point of the time plane was computed from −200 to 1,000 ms in the time domain and from 0.1 to 30 Hz in the frequency domain. The resulting spectrogram represented signal power as a joint function of time and frequency at each time–frequency point; this can be defined as:


$$\begin{array}{l} P\left(t,f\right)=|F\left(t,f\right){|}^{2} \end{array}$$

For each trial, the baseline interval was set from −200 to 0 ms. Baseline correction was achieved by the subtraction method in the time–frequency domain:


$$\begin{array}{l} P_{bl}\left(t,f\right)=P\left(t,f\;\right)-\bar{R}\left(f\right), \end{array}$$

where $\bar{R}\left(f\right)$ is the average power spectral density of the baseline interval at each frequency. A value of *P*_*bl*_(*t, f*) <0 was considered to represent ERD and a value of *P*_*bl*_(*t, f*) >0 was considered to represent ERS. ERD/ERS values for the theta (4–7 Hz), alpha (8–13 Hz), and beta (14–30 Hz) frequency bands at Fpz, FZ, CZ, and PZ were selected for analysis.

### 2.5. Statistical analysis

Participants' characteristics in the non-rhythmic vs. rhythmic condition were compared using paired *t*-tests, conducted using the statistical analysis software package SPSS (version 22). The significance level was judged according to the corresponding *p*-value; a *p*-value of < 0.05 was considered to represent statistical significance throughout all analyses.

## 3. Results

### 3.1. ERP results

**Figure 2:**
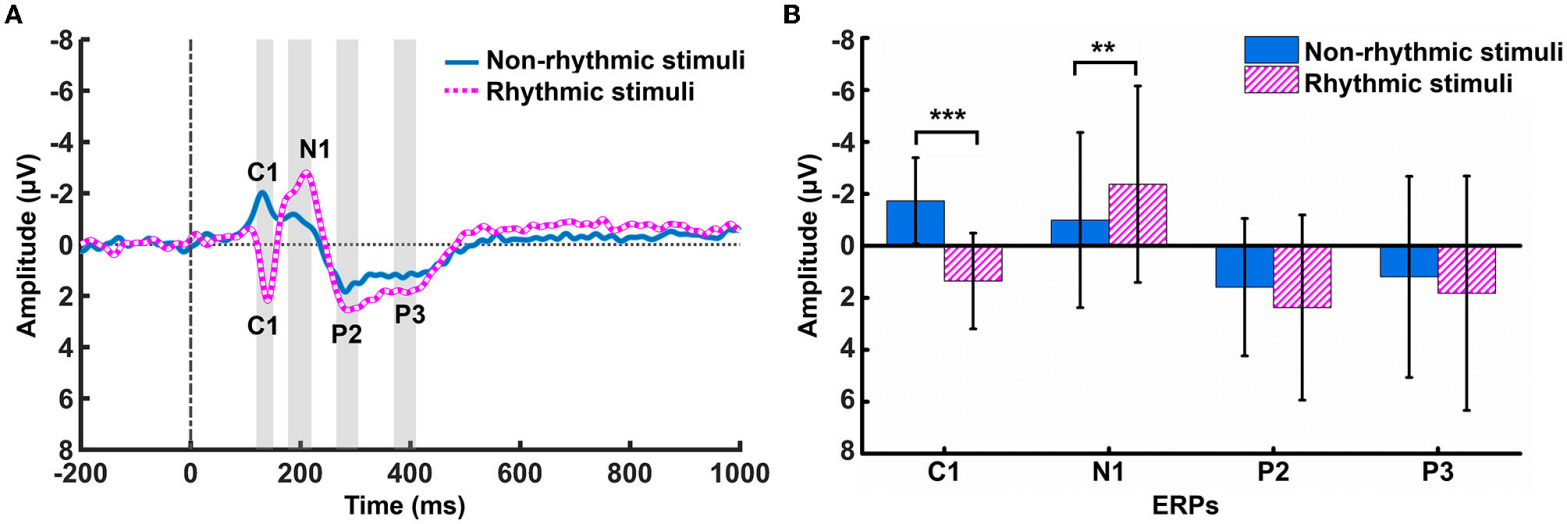
Differences in ERP amplitude at PZ under the non-rhythmic and rhythmic conditions. **(A)** The grand average ERP waveform over the parietal region (PZ) after cognitive processing of non-rhythmic (blue solid line) and rhythmic (pink dotted line) visual stimuli. C1, N1, P2, and P3 potentials appear after presentation of both types of visual stimuli. **(B)** Statistical comparison of the amplitudes of the C1, N1, P2, and P3 components of the ERP under the non-rhythmic and rhythmic conditions. *p < 0.05, **p < 0.01, ***p < 0.001.

There was no significant difference between the rhythmic and non-rhythmic conditions (t = 0.786, *p* = 0.443) in terms of the final number of trials entered into the analysis. Differences in amplitude changes occurring in ERPs over the parietal region (at the PZ electrode) in response to the non-rhythmic visual stimuli vs. rhythmic visual stimuli were examined. As shown in Figure 2, the C1, N1, P2, and P3 components of the ERP all formed under both types of visual stimulus. The C1 component was negative under non-rhythmic visual stimuli and positive under rhythmic visual stimuli. The results of a paired *t*-test showed that there was a significant difference between the two conditions in terms of the voltage of the C1 component (t = −6.043, *p* < 0.001). There was also a significant difference between the non-rhythmic and rhythmic conditions in the voltage of the N1 component (t = 3.060, *p* = 0.007). Compared with non-rhythmic visual stimuli, the amplitude of the N1 response to the rhythmic visual stimuli was higher. In terms of the P2 component, there was no significant difference between the non-rhythmic condition and the rhythmic condition (t = −1.232, *p* = 0.235). Similarly, for the P3 component, there was also no significant difference between the non-rhythmic condition and the rhythmic condition (t = −1.260, *p* = 0.225). However, for both the P2 and P3 components, the amplitude of the potential was more positive under exposure to rhythmic visual stimuli.

### 3.2. Microstate analysis results

**Figure 3:**
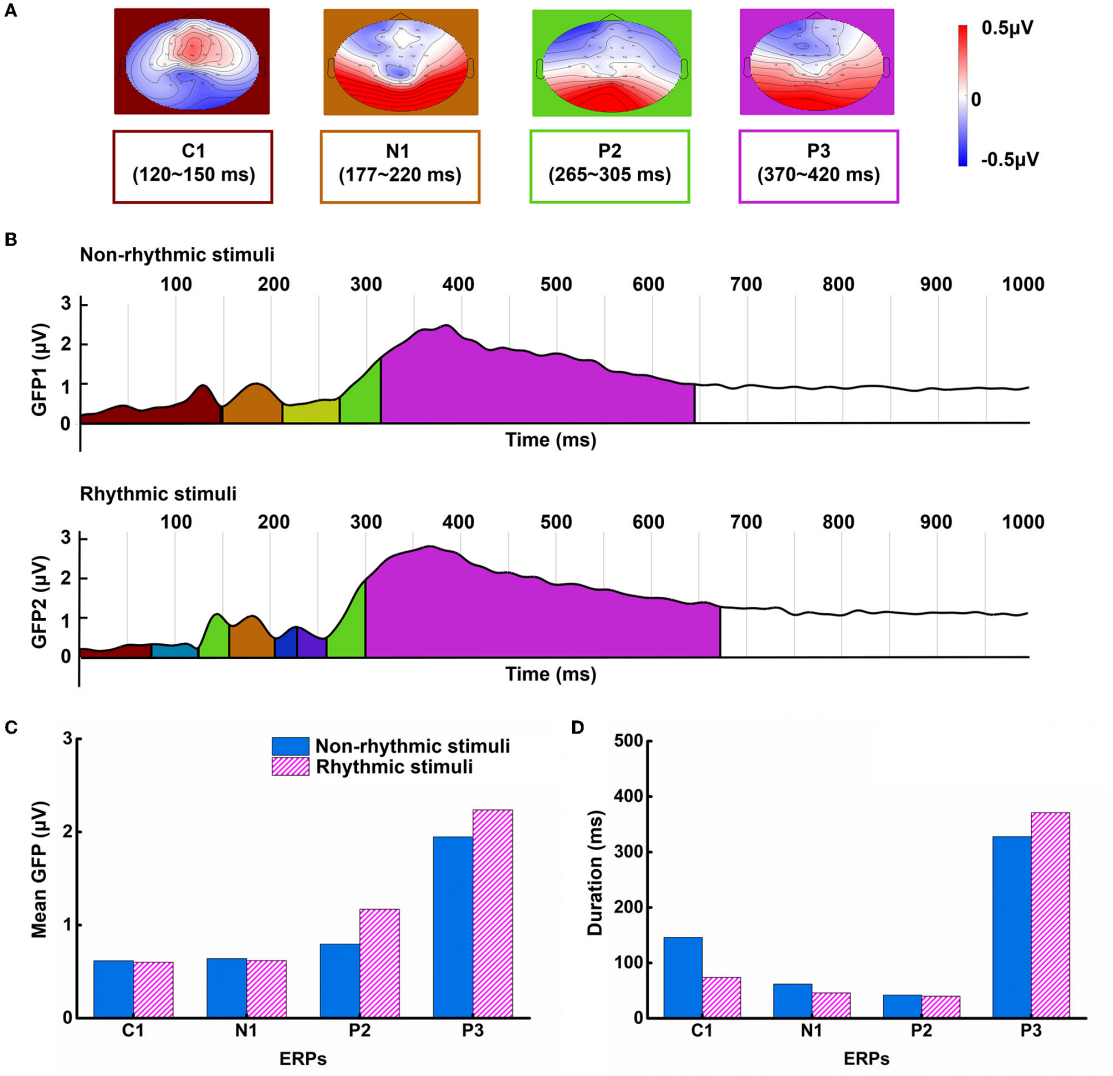
Differences in microstate patterns under the non-rhythmic and rhythmic conditions. **(A)** The topographical structure of four main microstate components. **(B)** Microstate GFP changes in visual cognitive processing under the non-rhythmic and rhythmic conditions. **(C)** The grand mean GFP of the four main microstate components under exposure to non-rhythmic stimuli and rhythmic stimuli. **(D)** The duration of the four main microstate components under exposure to non-rhythmic stimuli and rhythmic stimuli.

Based on the CV criterion, the results showed that the optimal microstate model consisted of 11 classes based on the ERP data of all participants and both stimulus conditions in the segment from 0 to 1,000 ms post-stimulus. Under the condition with non-rhythmic visual stimuli, there were seven main microstate categories; in comparison, under the condition with rhythmic visual stimuli, there were eight main microstate categories. Based on the changes in microstate function and their corresponding relationships with ERPs, we focused on the microstates associated with the C1, N1, P2, and P3 components. The microstate maps of the four ERPs, corresponding to the time range of the ERP peak, were obtained; these are shown in Figure 3A.

Figure 3B shows microstate assignment overlaid on a plot of GFP for the grand mean ERPs. The colors representing microstate assignment on the time axis match the colors used in the microstate topographic maps in Figure 3A. The black-brown portion represents GFP change corresponding to the C1 component; the brown portion represents GFP change corresponding to the N1 component; the green portion represents GFP change corresponding to the P2 component; and the violet portion represents GFP change corresponding to the P3 component. In addition to the above four main microstate categories, the results showed that additional subcomponents were present within the first 300 ms of visual processing under exposure to the rhythmic visual stimuli.

As shown in Figure 3C, the mean GFP and duration corresponding to each component were calculated for each of the two conditions. GFP was higher for the P3 component than for the other components under both conditions. Additionally, the GFP values for the P2 and P3 components were higher under exposure to the rhythmic visual condition than the non-rhythmic condition. Similarly, as shown in Figure 3D, the duration of the P3 component was the longest, and this component was longer under exposure to the rhythmic visual condition compared to the non-rhythmic visual condition.

### 3.3. ERD/ERS results

**Figure 4:**
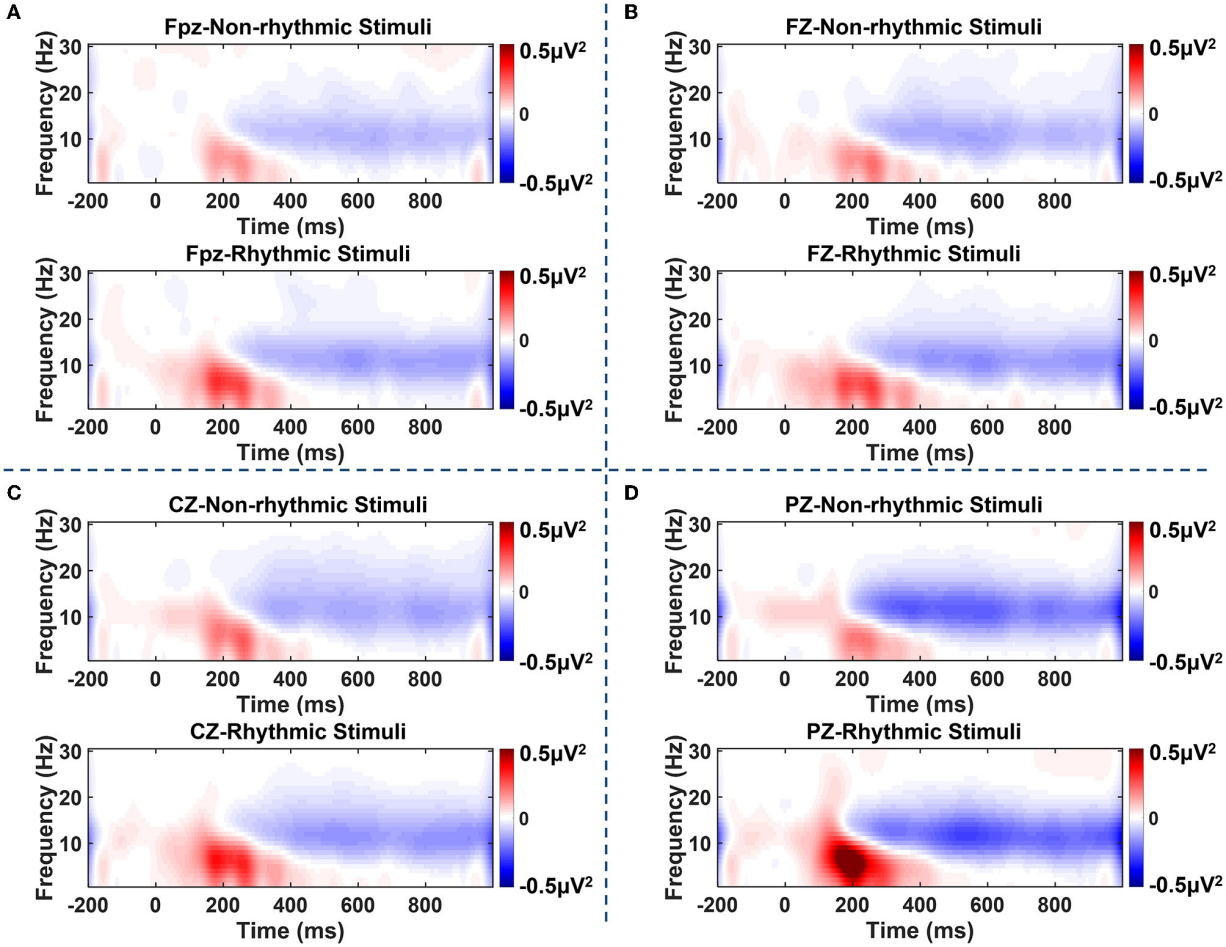
Time–frequency plots of amplitude recorded over brain regions at the Fpz, FZ, CZ, and PZ electrodes under non-rhythmic and rhythmic conditions. **(A–D)** Represent the time–frequency results for the FPz, FZ, CZ, and PZ electrodes, respectively, under exposure to non-rhythmic and rhythmic stimuli. Red areas in the time–frequency diagrams indicate the occurrence of ERS, and blue areas indicate ERD phenomena. The ERD/ERS phenomenon was most evident at the PZ electrode over the parietal region. Compared with non-rhythmic visual stimuli, the ERD/ERS phenomenon elicited by rhythmic visual stimuli was more evident at the midline electrode of all brain regions.

As shown in Figure 4, ERS of the theta band and alpha band could be observed over the electrodes located at the midline between 100 and 300 ms after the visual stimulus. After 300 ms, an obvious ERD phenomenon in the alpha band and beta band occurred at the midline electrodes over the prefrontal, frontal, central, and parietal regions. Specifically, the clearest occurrence of the ERD/ERS phenomenon occurred at the PZ electrode located in the parietal region under both the non-rhythmic and the rhythmic condition. Moreover, under exposure to the rhythmic visual stimuli, the ERD/ERS phenomenon was more obvious at the midline electrode of all brain regions compared with the non-rhythmic condition.

**Figure 5:**
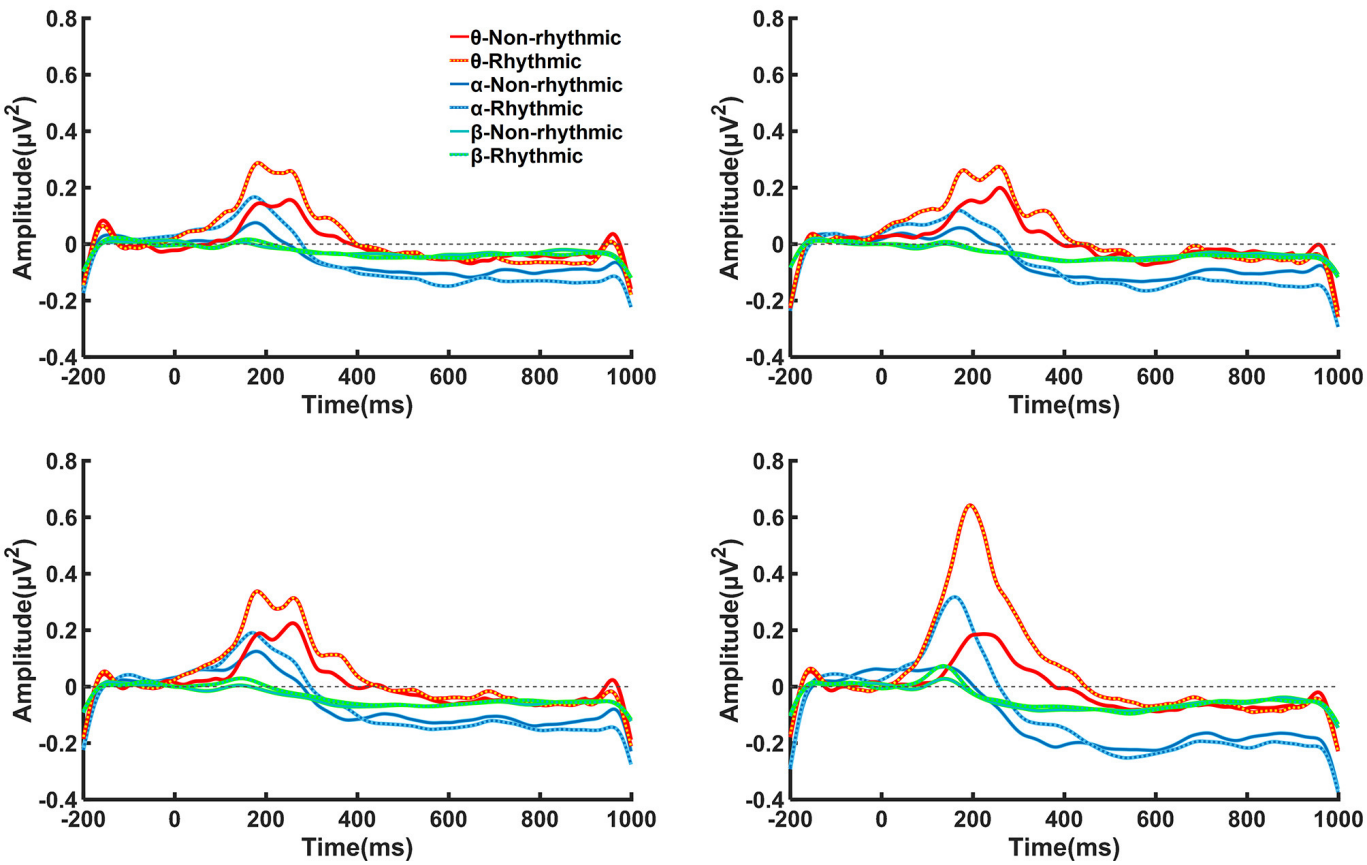
Time–frequency curves in the theta, alpha, and beta bands for brain regions recorded at the Fpz, FZ, CZ, and PZ electrodes under the non-rhythmic and rhythmic conditions. Transition processes from ERS to ERD in terms of power changes can be observed among the theta, alpha, and beta frequency bands. Under exposure to both the non-rhythmic and the rhythmic visual stimuli, power changes in the theta, alpha, and beta frequency bands mainly occurred during the period of 100–600 ms. For the theta frequency band, θ-ERS was dominant during the period of 100–400 ms after onset of the visual stimulus, while θ-ERD was not clear.

On this basis, we analyzed power changes at the four electrodes at the midline in different frequency bands. As shown in Figure 5, there were transition processes from ERS to ERD in terms of power changes observed among the theta, alpha, and beta frequency bands. Under exposure to both the non-rhythmic and the rhythmic visual stimuli, the power changes in the theta, alpha, and beta frequency bands mainly occurred in the period of 100–600 ms. For the theta frequency band, θ-ERS was dominant during the period 100–400 ms after the visual stimulus, while θ-ERD was not clear. For the alpha frequency band, α-ERS mainly manifested during the period 100–300 ms after visual stimuli; α-ERD was also observed after 300 ms of exposure to the visual stimulus. For the beta frequency band, the β-ERS phenomenon was hardly observed. A β-ERD phenomenon was observed after 200 ms of exposure to the visual stimulus, but its power was lower than that of α-ERD.

**Figure 6:**
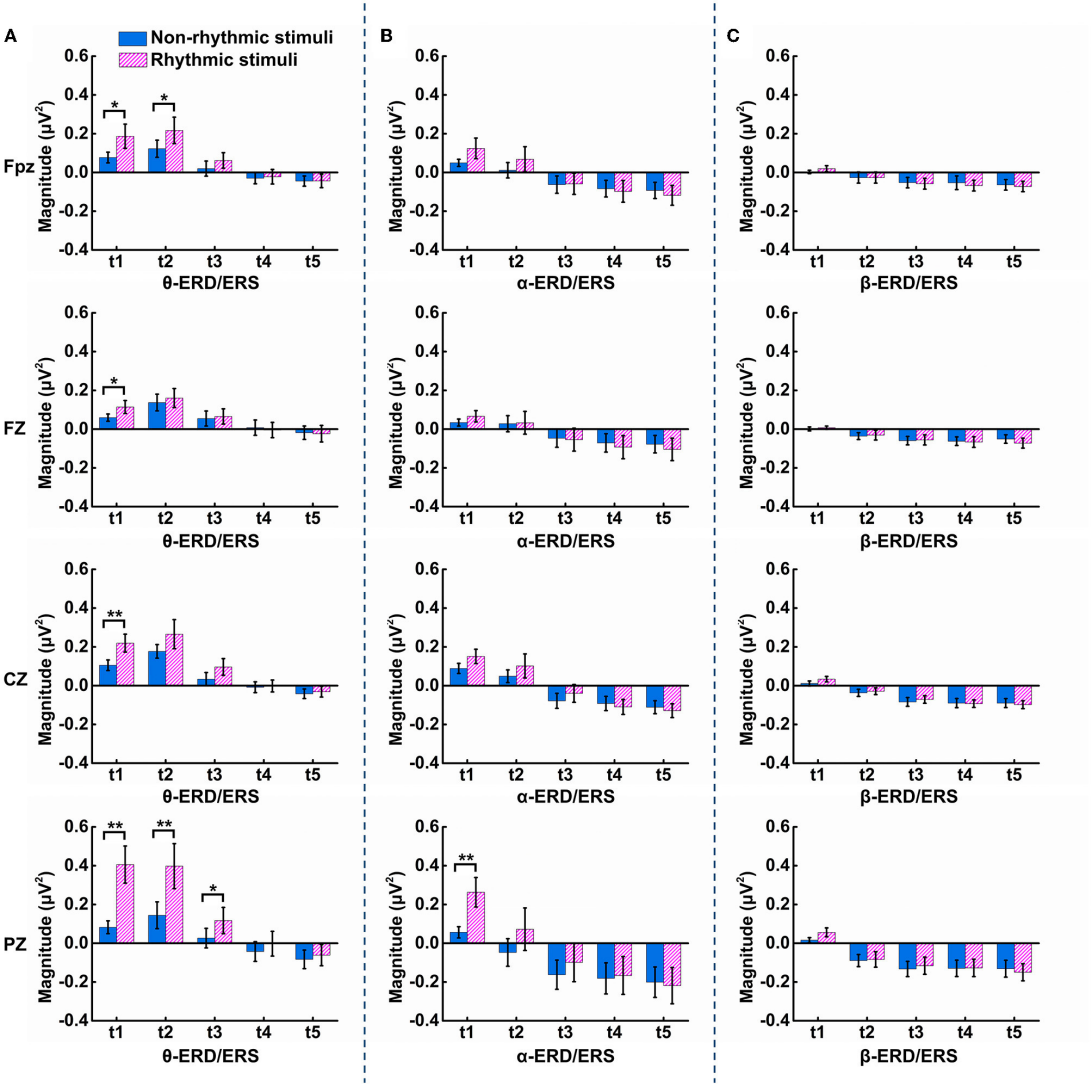
Results of the statistical comparison of ERS/ERD at the Fpz, FZ, CZ, and PZ electrodes in the theta, alpha, and beta bands under the non-rhythmic and rhythmic conditions. **(A)** Statistical differences in θ-ERS/ERD between the non-rhythmic and rhythmic conditions. **(B)** Statistical differences in α-ERS/ERD between the non-rhythmic and rhythmic conditions. **(C)** Statistical differences in β-ERS/ERD between the non-rhythmic and rhythmic conditions.

Statistical analysis of ERD/ERS was carried out for the three frequency bands of theta, alpha, and beta at the midline electrodes over each brain region. As shown in Figure 6A, within the t1 period (100–200 ms after onset of the visual stimulus), there were significant differences in terms of θ-ERS between the non-rhythmic and rhythmic conditions at the midline electrodes over the prefrontal (Fpz: t = −2.768, *p* = 0.013), frontal (FZ: t = −2.604, *p* = 0.019), central (CZ: t = −2.989, *p* = 0.008), and parietal (PZ: t = −4.033, *p* = 0.001) regions. θ-ERS was significantly stronger under exposure to the rhythmic visual stimuli than under the non-rhythmic visual stimuli in the t1 period for all midline electrodes, especially the PZ electrode over the parietal region. In addition, within the t2 period (200–300 ms after onset of the visual stimulus), there were also significant differences in terms of θ-ERS between the two conditions at the electrodes over the prefrontal (Fpz: t = −2.332, *p* = 0.032) and parietal regions (PZ: t = −3.670, *p* = 0.002). Similarly, θ-ERS was significantly stronger under exposure to the rhythmic condition than under exposure to its non-rhythmic counterpart within the t2 period at both the Fpz and PZ electrodes. Within the t3 period (300–400 ms after onset of the visual stimulus), θ-ERS was more evident under exposure to the rhythmic condition, although a significant difference was observed only at the PZ electrode (PZ: t = −2.796, *p* = 0.012). For the alpha frequency band, a significant difference between the non-rhythmic and rhythmic conditions was observed only at the PZ electrode during the t1 period, as shown in Figure 6B (PZ: t = −3.259, *p* = 0.005). In response to the rhythmic visual stimuli, α-ERS was significantly stronger during t1 than in response to their non-rhythmic counterparts. As shown in Figure 6C, there was no significant difference in ERD/ERS for the beta band at any of the midline electrodes over the entire brain area.

**Figure 7:**
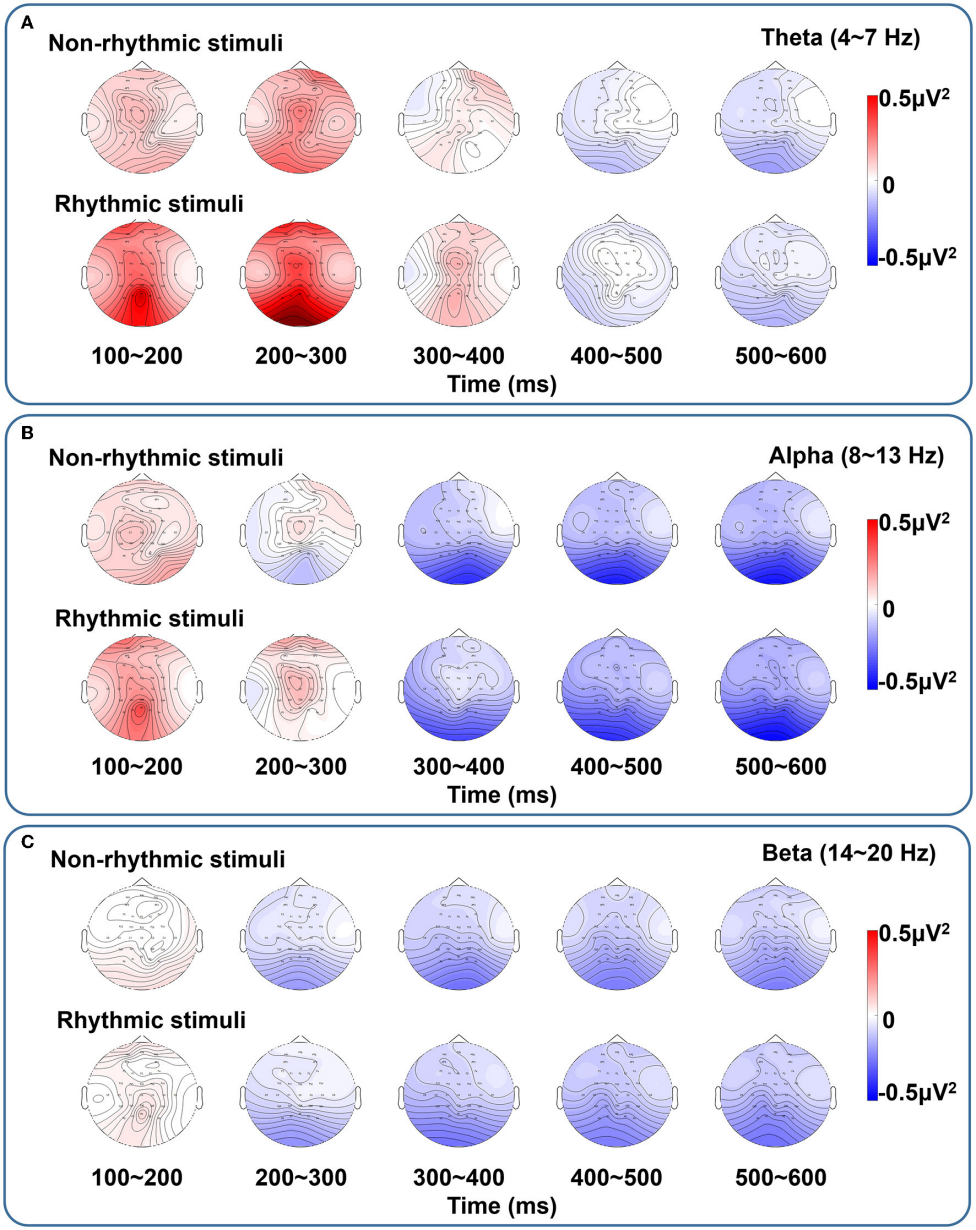
Brain topographic maps of spectral characteristics in the theta, alpha, and beta bands under non-rhythmic and rhythmic conditions. **(A–C)** Represent the brain topographic maps in the theta, alpha, and beta bands, respectively, under exposure to non-rhythmic and rhythmic stimuli. The ERD/ERS process at all electrodes over all brain regions in response to the non-rhythmic visual stimuli and rhythmic visual stimuli is shown. In the period of 100–300 ms, ERS is dominant in the theta band the non-rhythmic and rhythmic conditions. In addition, ERS in the theta band seems to be more evident under the rhythmic condition. In the period of 300–600 ms, ERD in the alpha band is dominant under both conditions. It seems that there is no clear difference between the two conditions. For the beta band, ERD is present mainly in the period of 200–600 ms, but ERD in the beta band is weak under both the non-rhythmic and rhythmic conditions. Similarly, there is again no clear difference between the two conditions.

Figure 7 shows brain topographic maps of the spectral characteristics in the theta, alpha, and beta frequency bands under the non-rhythmic and rhythmic conditions. ERD/ERS at all electrodes over all brain regions in response to the non-rhythmic visual stimuli and the rhythmic visual stimuli are shown. During the period of 100–300 ms, ERS of the theta band was dominant under the non-rhythmic and rhythmic conditions. In addition, ERS of the theta band seemed to be more evident under the rhythmic condition. In the period of 300–600 ms, ERD of the alpha band was dominant under the two conditions. It seemed that there was no obvious difference between the two conditions. Finally, for the beta band, ERD was present mainly in the period of 200–600 ms, but ERD of the beta band was weak under both the non-rhythmic and rhythmic conditions. Similarly, there was again no clear difference between the two conditions.

## 4. Discussion

We performed a multimodal analysis of the processes involved in cognitive processing of rhythmic visual information and compared the EEG characteristics of the time domain, space–time domain, and time–frequency domain under exposure to rhythmic visual stimuli and non-rhythmic visual stimuli in order to analyze the characteristics of the distribution of cognitive resources under rhythmic visual stimulation. First, we found that the C1 component was positive under exposure to rhythmic visual stimuli, and the amplitude of the N1 component was greater under exposure to rhythmic visual stimuli as compared to their non-rhythmic counterparts. Second, more microstate categories occurred under exposure to rhythmic visual stimuli. The grand GFP amplitude of the P3 microstate component was greater under exposure to rhythmic visual stimuli compared to non-rhythmic visual stimuli, and the duration of the P3 microstate component was also longer under exposure to rhythmic visual stimuli. Third, compared with the non-rhythmic condition, ERS of the theta band was more evident under exposure to rhythmic visual stimuli at all analyzed brain regions within the first 200 ms of input. There was no significant difference in either alpha ERD or beta ERD between the non-rhythmic and rhythmic conditions. Overall, these findings suggest that the demands for cognitive resources in response to visual processing were dynamic before gait-related motor initiation, and the modulation of allocation of cognitive resources was clearer in the case of rhythmic visual processing than in that of non-rhythmic visual processing.

### 4.1. Methodological considerations regarding representation of cognitive resources

The human brain is flexible in the allocation of cognitive resources, which can be allocated to important new stimuli according to task requirements. The more complex a stimulus or processing task is, the more cognitive resources need to be recruited. When all cognitive resources are entirely occupied, new stimuli will not be processed, and cognitive functions will be limited according to the cognitive needs of the task (Cohen et al., 2012). Therefore, clarifying the method of representation of cognitive resources will help us to understand cognitive processing under different visual stimuli. By analyzing the electrophysiological signals captured by EEG in different dimensions, we can promote a comprehensive understanding of the allocation of cognitive resources.

In examining cognitive processing under exposure to different visual stimuli, this study first analyzed the ERP distribution in the parietal region through EEG time-domain analysis. As the parietal region is closely related to visual information perception and processing, the distribution of ERPs in this brain region deserves special attention. The results showed that the C1, N1, P2, and P3 ERP components appeared successively at the PZ electrode of the parietal region under exposure to both non-rhythmic and rhythmic visual stimuli. These ERP components represent visual field input, visual information perception, and early and late cognitive processing of visual stimuli, respectively (Luck et al., 2000; Duncan et al., 2009; Hickey et al., 2020). The amplitude difference in these ERP components could be used as an evaluation index for resource recruitment evaluation in terms of the cognitive processing of non-rhythmic and rhythmic visual stimuli.

In addition, this study innovatively assessed the brain network dynamics of cognitive processing of non-rhythmic and rhythmic visual stimuli through microstate analysis. From the perspective of the space–time domain, each spatial extension structure can be taken to represent a microstate category, representing different cognitive processing modes (Michel and Koenig, 2018). Under exposure to both non-rhythmic and rhythmic visual stimuli, the main microstate categories were essentially consistent with the components observed in the ERP analysis, reflecting the main modes of visual cognitive processing. Moreover, there were differences in the number of microstate categories that emerged under each of the two conditions. The more microstate categories occurred, the greater the resources needed to coordinate the formation of different activation states over the entire brain. Furthermore, the duration and GFP peak of a particular microstate category can be taken to represent the consumption of resources. Therefore, we examined the differences in cognitive resources by analyzing the categories, the duration, and the GFP of each microstate under exposure to non-rhythmic and rhythmic visual stimuli.

Previous studies have shown that ERPs elicited during motor preparation can predict individual oscillatory performance (Tomassini et al., 2017). That is, there is a correlation between ERPs and spectrum oscillation. ERD and ERS phenomena in the time–frequency domain may reflect the characteristics of oscillation of cortical activation in cognitive processing. These phenomena can also be used as important indicators of cognitive resource consumption. Through time–frequency analysis of the prefrontal, frontal, central, and parietal regions, this study found that ERS of theta oscillation differed significantly between the conditions with non-rhythmic and rhythmic visual stimuli. This showed that ERS of theta oscillation was sensitive to the recruitment of cognitive resources.

In summary, this study evaluated EEG characteristics on multiple dimensions, such as time-domain, space–time domain, and time–frequency domain characteristics. This can enable more comprehensive representation of the allocation of cognitive resources. This approach was helpful in clarifying the effects of cognitive processing under exposure to rhythmic visual stimuli on the allocation of cognitive resources.

### 4.2. Allocation of cognitive resources in visual cognitive processing

In terms of ERPs, there were significant differences in cognitive processing under exposure to non-rhythmic vs. rhythmic visual stimuli for the C1 component and the N1 component. Under exposure to rhythmic visual stimuli, the C1 component was positive, indicating that participants' visual processing focused mainly on the lower visual field. Under exposure to non-rhythmic visual stimuli, the C1 component took a negative value, indicating that participants' visual processing focused mainly on the upper visual field (Luck et al., 2000). That is, rhythmic visual cues induced participants to move their attention down the visual field when processing visual information. Research has shown that visual processing consumes less cognitive resources when it is focused on the lower portion of the visual field. As an ERP component related to early processing in visual perception, the higher the amplitude of the N1 component, the greater the cognitive processing demands (Gramann et al., 2010). The results showed that the amplitude of N1 was higher under the rhythmic condition than that under the non-rhythmic condition, indicating that perceptual processing of rhythmic visual stimuli consumed more cognitive resources. Compared with processing of non-rhythmic visual information, processing of the visual field input within the first 200 ms of rhythmic visual stimulation consumed fewer resources, while there were more demands on cognitive resources in subsequent visual perception and cognitive processing under exposure to rhythmic visual stimuli.

There was an obvious correlation between the microstate categories and ERP components. Different topographical topologies are generated by the coordinated activities of different neural combinations in the brain. Transformations between microstate categories can be interpreted as representing the sequential activation of different neural networks (Michel and Koenig, 2018). In the case of cognitive processing of rhythmic visual stimuli, the presence of more microstate categories indicated a more complex brain function network topology (Khanna et al., 2015). In microstate analysis, GFP peak and average duration can be taken to characterize the stable state of each ERP component (Rohde et al., 2020). Comparing the four microstate categories that corresponding to ERP components, the changes in GFP and average duration under exposure to visual stimuli showed that the cognitive processing of visual stimuli mainly occurs during the period of the P3 component. This shows that late cognitive processing consumed more cognitive resources in the case of exposure to rhythmic visual stimuli compared with non-rhythmic visual stimuli.

The distribution of spectrum oscillation in the brain plays an important role in motor coordination and cognitive processing (Yordanova et al., 2020). θ-ERS modulation has been interpreted as representing the level of resource allocation in sensorimotor processing (Fiebelkorn and Kastner, 2019; Benedetto et al., 2021). The stronger θ-ERS observed under exposure to rhythmic visual stimuli showed that rhythmic visual stimuli consume fewer cognitive resources within the first 200 ms of input. ERD occurred with a temporal change in cognitive processing, and the consumption of cognitive resources increased gradually.

α-ERD is associated with cortical inhibition (Hunter, 2020). The power of alpha rhythm reflects active neuronal processing; that is, strong α-ERD is more likely to reflect a high demand for attention resources (Yang et al., 2020). Although there was no statistically significant difference in the ERD amplitude at the alpha band between the non-rhythmic and rhythmic conditions, α-ERD under exposure to rhythmic visual stimuli appeared to be a little stronger than under exposure to non-rhythmic visual stimuli. According to our analysis, this lack of statistical significance could have arisen for two possible reasons. One reason could be that the number of subjects was insufficient to make the difference statistically significant. The other possibility is that, for healthy subjects, differences in their cognitive processing under exposure to the two types of visual stimuli were relatively small.

The power of the beta rhythm reflects the level of motor preparation (Tzagarakis et al., 2015). Under both the non-rhythmic and rhythmic conditions, the relatively weak β-ERD phenomenon indicated that the stage that we analyzed was mainly related to visual cognitive processing, and the stage of motor preparation had not been entered. Consistent with the results of previous research, the ERS/ERD phenomenon was most prominent in the parietal region, which indicates that the processing of visual information mainly occurs in the parietal region.

### 4.3. The relationship between allocation of cognitive resources and gait-related motor initiation

The performance of human cognitive and motor functions largely depends on the allocation of brain resources in synchronous tasks (Vila-Cha et al., 2021). Under increasing task demands, maintaining adequate motor ability depends on the regulation and coordination of cognitive resources. Previous studies have also proposed the possibility that cognitive load and the allocation of cognitive resources may affect the motor model (Hong et al., 2014). Based on the hypothesis that cognitive and motor functions draw on a common neural resource pool, it is believed that an increase in cognitive load may lead to motor incoordination (Stern, 2002). Individuals with cognitive impairment need to use more neural circuits to complete less demanding cognitive tasks and therefore need to draw on “cognitive reserves” to a greater extent (Scarmeas et al., 2003; Balart-Sanchez et al., 2021); thus, they are constrained by cognitive resources and their task performance is affected (Yang et al., 2020). Generally speaking, short supply of cognitive resources may lead to motor disability.

For patients with PD, the decrease in dopaminergic secretion in the basal ganglia leads to a shortage of related cognitive resources (Marquez et al., 2020). Therefore, we speculate that this lack of cognitive resources may be the cause of FOG in PD patients, resulting in their inability to proceed with GI normally. One hypothesis is that rhythmic visual cues may reduce the demands on cognitive resources for FOG patients in completing GI.

Based on this hypothesis, we conducted a series of studies on the effect of rhythmic visual cues on the allocation of cognitive resources in healthy subjects. In our previous study, we found that exposure to rhythmic visual cues could reduce cognitive resource demands on healthy subjects during motor preparation (Zhou et al., 2021). Extending this work, the present study focused on visual cognitive processing of rhythmic visual stimuli. We found that, under exposure to rhythmic visual stimuli, demands for cognitive resources were lower during the early stages of input of visual information and gradually increased during visual perception and subsequent visual cognitive processing. Linking the findings of the two studies, from visual cognitive processing to motor preparation, rhythmic visual cues may exert a better regulatory effect on the allocation of cognitive resources for normal healthy subjects in completion of GI.

### 4.4. Study limitations and future research

The long-term goal of our series of studies is to reveal the mechanism by which rhythmic visual cues produce improvement in GI for FOG patients with PD. Based on the summary of relevant literature and clinical phenomena in the field, this study first carried out the EEG characteristic analysis of rhythmic visual stimuli in normal subjects. However, there were no relevant data on PD patients. Therefore, whether the research conclusion of this study could be extended to PD patients needs to be discussed in further relevant research, which will be carried out on PD patients in the future.

## 5. Conclusion

To our knowledge, this is the first report that confirms the characteristics of the dynamic allocation of cognitive resources in rhythmic visual cognitive processing and provides some neurophysiological basis for the findings via analysis of EEG. The results of ERP, ERS/ERD, and microstate analysis all showed that consumption of cognitive resources was lower within the first 200 ms under exposure to rhythmic visual information, while in the subsequent stages of visual cognitive processing, the demand for cognitive resources was higher under exposure to rhythmic visual stimuli. Based on the results of this study, due to the higher level of cognitive processing of rhythmic visual information, exposure to this type of cue can be more conducive to completion of GI-related motor preparation activities in the later period. Therefore, there is less demand for cognitive resources during preparation of GI. Thus far, research on the allocation of cognitive resources during the cognitive processing of rhythmic visual stimuli and GI preparation has demonstrated that the dynamic allocation of cognitive resources is key to improving gait-related movement based on rhythmic visual cues. This study has not been carried out with PD patients. Further research needs to be carried out with PD patients in order to verify whether the conclusions of this study are applicable to these patients.

## Data availability statement

The original data and materials presented in this article can be obtained from the corresponding authors upon request.

## Ethics statement

The studies involving human participants were reviewed and approved by Ningbo Institute of Materials Technology and Engineering, Chinese Academy of Sciences. The patients/participants provided their written informed consent to participate in this study.

## Author contributions

GZ: conceptualization, methodology, writing—review and editing, and funding acquisition. CS: conceptualization, methodology, writing—review and editing, supervision, and funding acquisition. HZ: conceptualization, methodology, software, validation, formal analysis, investigation, resources, writing—original draft, visualization, and funding acquisition. WY: formal analysis and investigation. JX: software, supervision, and funding acquisition. YM: formal analysis. All authors contributed to the article and approved the submitted version.
